# Bilateral international migration flow estimates for 200 countries

**DOI:** 10.1038/s41597-019-0089-3

**Published:** 2019-06-17

**Authors:** Guy J. Abel, Joel E. Cohen

**Affiliations:** 1 Asian Demographic Research Institute, Shanghai University, Shanghai, China; 2 Wittgenstein Centre (IIASA, VID/OEAW, WU) International Institute for Applied Systems Analysis, Laxenburg, Austria; 3 Laboratory of Populations, The Rockefeller University and Columbia University, New York, NY 10065 USA; 4 Earth Institute and Department of Statistics, Columbia University, New York, NY 10027 USA; 5 Department of Statistics, University of Chicago, Chicago, IL 60637 USA

**Keywords:** Geography, Social sciences

## Abstract

Data on stocks and flows of international migration are necessary to understand migrant patterns and trends and to monitor and evaluate migration-relevant international development agendas. Many countries do not publish data on bilateral migration flows. At least six methods have been proposed recently to estimate bilateral migration flows between all origin-destination country pairs based on migrant stock data published by the World Bank and United Nations. We apply each of these methods to the latest available stock data to provide six estimates of five-year bilateral migration flows between 1990 and 2015. To assess the resulting estimates, we correlate estimates of six migration measures from each method with equivalent reported data where possible. Such systematic efforts at validation have largely been neglected thus far. We show that the correlation between the reported data and the estimates varies widely among different migration measures, over space, and over time. We find that the two methods using a closed demographic accounting approach perform consistently better than the four other estimation approaches.

## Abstract (summary)

**Table:** 

Design Type(s)	modeling and simulation objective • population data analysis objective • data validation objective
Measurement Type(s)	Human Migration
Technology Type(s)	computational modeling technique
Factor Type(s)	geographic location • temporal_interval
Sample Characteristic(s)	Homo sapiens • Earth (Planet) • populated place

Machine-accessible metadata file describing the reported data (ISA-Tab format)

## Background & Summary

International migration is becoming an ever more important component of population growth^1^, a driver for socio-economic change^2,3^ and a topic for policy debate^4^ in many countries. Good data on international migration are becoming more important as a means to monitor international development agendas and agreements such as the Sustainable Development Goals and Global Compact for Safe, Orderly and Regular Migration^5^. The estimates of migration flows are of potential use to those studying global or regional migration systems, such as economists and sociologists who study migration drivers and networks, demographers and climate change scientists for base line data for projections, and epidemiologists who study the role of human movements in the transmission of infectious diseases.

However, reliable international migration flow data, to measure the movement of people between countries over a given period, are scarce^6^. In many countries, migration flow data are not collected because data collection systems are expensive. Countries that collect migration flow data often use widely varying criteria for defining migrants (such as their length of stay and purpose for moving). These definitional differences prevent detailed cross-national comparisons. Combined, missing and non-comparable data prohibit accurate measurement of patterns and trends in global migration flows.

The lack of accurate migration flow data has motivated recent efforts to develop synthetic estimates for bilateral flows between all pairs of countries^7–11^. Each of these methods relies on bilateral migrant stock data. Migrant stock data measure the number of migrants at a particular point in time residing in each country by their country of birth. Such measures are easier to collect than flows, requiring only birthplace questions from a country’s census, population register or administrative data collection system.

Currently proposed methods to estimate migration flows from changes in migrant stocks fall into three groups. The first set of methods uses the differences in successive bilateral stocks for a given pair of countries to estimate the corresponding migration flows in each direction. These approaches are commonly used by economists and regional scientists who use these migration flow estimates as a dependent variable in their explanatory models. The second group of methods uses migrant stock data to estimate migration flow rates, which are then multiplied by a population at risk. The third group of methods frames the changes in migrant stocks as the residuals in a global demographic account. These so-called “demographic accounting” approaches estimate migration flows to match increases or decreases in the reported bilateral stocks of migrants, and births and deaths during the period.

Thus far, the different estimation methods have used different migrant stock data as inputs, prohibiting a systematic comparison. Many have not used newly published migrant stock and demographic data. Further, systematic comparisons of different sets of estimates are lacking. Previous applications of the estimation methods typically do not compare outputs with either results from alternative methods or reported data. Where comparisons are undertaken, they are performed for only a small fraction of pairs of countries. This lack of comparison has made it difficult for users to know which estimation method produces values most relevant to their application.

To supply migration flow estimates based on the most recent migrant stock data and systematic comparisons of different methods, in this paper we apply multiple methods for estimating migration flows from stocks to produce estimates of the bilateral international migration flows between all pairs of 200 countries for five five-year periods from mid-year (July 1) 1990 to mid-year (June 30) 2015, using the most recent set of bilateral migration stocks published by the United Nations. We also propose a set of validation exercises based on a number of commonly used migration measures and reported migration flow data. These comparisons highlight the strengths and weaknesses of different sets of estimates.

**Fig. 1:**
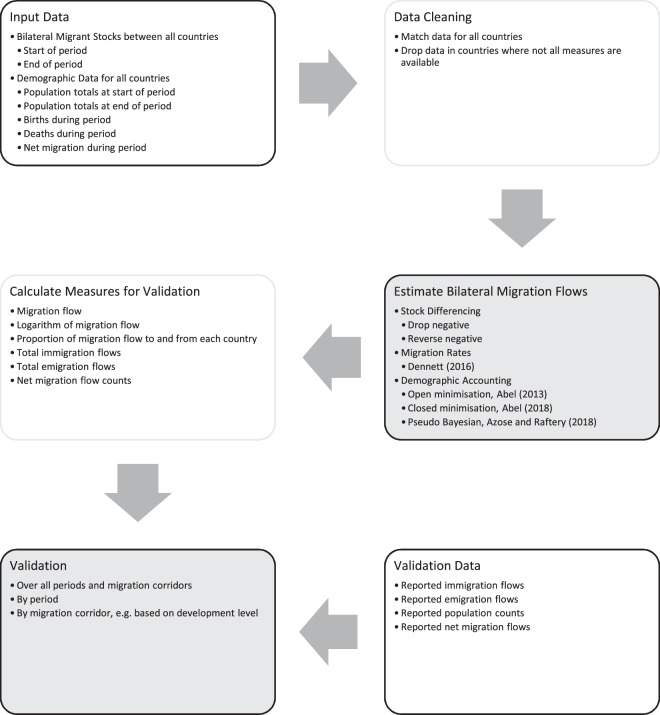
Overview of study design for estimation and validation of bilateral migration flows between all countries. Data for input and comparison shown in black and white. Data processing steps shown in boxes with shaded outlines. Resulting data shown boxes with shaded background.

An overview of our study design is shown in Fig. 1. The following sections outline the estimates of bilateral international migration between all pairs of 200 countries for five-year periods during 1990–2015 based on six estimation methods.

## Methods

In this section we outline six methods. We use the same notation and a simplified hypothetical data set, introduced next, to allow for easier comparison between the methods. Details on each estimation method are provided towards the end of this section, before an overview of the code used to perform our calculations.

### Contingency tables

Bilateral migration data are commonly represented in square contingency tables with off-diagonal entries (the cell in the intersection of row *i* and column *j*, *i* ≠ *j*) containing a migration measure from origin *i* to destination *j*. Consider two hypothetical migrant stock tables in consecutive years (*t* and *t* + 1) in Table 1. Regions A to D represent places of birth in the rows, and place of residence in the columns. The off-diagonal entries represent the number of foreign-born migrants in each area of residence, while the diagonal entries contain the number of native-born residents.

**Table 1:** Hypothetical bilateral migrant stock data. On the left side are migrant stock data at time *t*. On the right side are migrant stock data at time *t* + 1.

Birthplace	Place of Residence (*t*)					Birthplace	Place of Residence (*t* + 1)				
	A	B	C	D	Sum		A	B	C	D	Sum
A	100	10	10	0	120	A	95	5	15	5	120
B	80	180	10	90	360	B	75	225	5	55	360
C	30	10	140	40	220	C	55	0	115	50	220
D	60	70	10	160	300	D	35	25	25	215	300
Sum	270	270	170	290	1000	Sum	260	255	160	325	1000

### Stock differencing

#### Stock difference, drop negative

One way to estimate bilateral migration flows from migrant stock tables is to find the difference in the size of migrant stocks at the beginning and end of a given period. The simplest form of differencing to estimate the number of migrant transitions *y*_*ij*_ from origin *i* to destination *j* during a unit time interval subtracts the migrant stock totals in each migration corridor (a one-directional flow from *i* to *j* between a pair *i*, *j* of countries), setting all negative differences to zero:

$${y}_{ij}=\{\begin{array}{cc}{s}_{ij}^{t+1}\,-\,{s}_{ij}^{t} & {\rm{i}}{\rm{f}}\,{s}_{ij}^{t+1} > {s}_{ij}^{t}\,{\rm{a}}{\rm{n}}{\rm{d}}\,i\ne j,\\ 0 & {\rm{i}}{\rm{f}}\,{s}_{ij}^{t+1}\le {s}_{ij}^{t}\,{\rm{o}}{\rm{r}}\,i=j,\end{array}$$

where ${s}_{ij}^{t}$ and ${s}_{ij}^{t+1}$ refer to the number of migrants born in *i* and residing in *j* at time *t* and *t* + 1 respectively and *i* = 1,2, …, *R* and *j* = 1,2, …, *R* for *R* places of birth and residence.

This method has been used to create a dependent variable for econometric models to explain migration flows^12,13^. The left side of Table 2 illustrates the migration flows estimated from applying this method to the hypothetical migrant stock data in Table 1.

**Table 2:** Estimated bilateral migration flows from stock differencing. On the left side are the estimated migration flows when negative differences are set to zero. On the right side are the estimated migration flows when negative stock differences are reversed. Diagonal elements are all zero.

Stock Differencing - Drop Negatives						Stock Differencing - Reverse Negatives					
Origin (*t*)	Destination (*t* + 1)					Origin (*t*)	Destination (*t* + 1)				
	A	B	C	D	Sum		A	B	C	D	Sum
A		0	5	5	10	A		5	5	30	40
B	0		0	0	0	B	5		10	45	60
C	25	0		10	35	C	25	5		10	40
D	0	0	15		15	D	0	35	15		50
Sum	25	0	20	15	60	Sum	30	45	30	85	190

#### Stock difference, reverse negative

Beine and Parsons (2015)^14^ treated decreases in bilateral migrant stocks as a reverse migration flow from origin *j* to destination *i*:

$${y}_{ij}=\left\{\begin{array}{ll}{s}_{ij}^{t+1}\,-\,{s}_{ij}^{t} & {\rm{if}}\,{s}_{ij}^{t+1} > {s}_{ij}^{t}\,{\rm{and}}\,{s}_{ji}^{t+1}\ge {s}_{ji}^{t}\,{\rm{and}}\,i\ne j,\\ {s}_{ij}^{t+1}\,-\,{s}_{ij}^{t}+{s}_{ji}^{t}\,-\,{s}_{ji}^{t+1} & {\rm{if}}\,{s}_{ij}^{t+1} > {s}_{ij}^{t}\,{\rm{and}}\,{s}_{ji}^{t+1} < {s}_{ji}^{t}\,{\rm{and}}\,i\ne j,\\ {s}_{ji}^{t}\,-\,{s}_{ji}^{t+1} & {\rm{if}}\,{s}_{ij}^{t+1}\le {s}_{ij}^{t}\,{\rm{and}}\,{s}_{ji}^{t+1} < {s}_{ji}^{t}\,{\rm{and}}\,i\ne j,\\ 0 & {\rm{if}}\,{s}_{ij}^{t+1}\le {s}_{ij}^{t}\,{\rm{and}}\,{s}_{ji}^{t+1}\ge {s}_{ji}^{t}\,{\rm{or}}\,i=j.\end{array}\right.$$

The estimated migration flows following this method for the hypothetical migrant stock data in Table 1 are shown on the right side of Table 2.

### Migration rates

Dennett (2016) proposed a method for estimating global bilateral migration flows using a migrant stock table at a single time point and a measure of total global migration flows^10^. The migration stock table is used to estimate migration flow rates over a specified period by dividing all migrant stock counts (in the off-diagonal cells) by the global foreign-born population. These rates are then multiplied by additional data on the estimated global migration flows *M*:

$${y}_{ij}=\{\begin{array}{cc}M\frac{{s}_{ij}^{t}}{{{\rm{\Sigma }}}_{gh,g\ne h}{s}_{gh}^{t}} & {\rm{i}}{\rm{f}}\,i\ne j\\ 0 & {\rm{i}}{\rm{f}}\,i=j\end{array}$$

As the total *M* is unknown, Dennett (2016) suggested approximating it using the sum of absolute net migration flows for the time interval. The hypothetical data in Table 1 are constructed on the assumption that births minus deaths equal zero in each country over the time interval, hence in both tables the global sum of migrant stocks is the same. Further, the sum of absolute net migration flows for the time interval can be derived as the difference in the population totals (the column totals) in each place of residence. Hence, $M={{\rm{\Sigma }}}_{j=A}^{D}|{{\rm{\Sigma }}}_{i=A}^{D}{s}_{ij}^{t+1}-{{\rm{\Sigma }}}_{i=A}^{D}{s}_{ij}^{t}|=|270-260|+|270-255|+|170-160|+|325-290|$ = 10 + 15 + 10 + 35 = 70 can be applied to estimate the bilateral migration flows shown in Table 3. Table 1 gives ${{\rm{\Sigma }}}_{g,h,g\ne h}{s}_{gh}^{t}=420$, hence the flow *rate* from B to A is estimated to be $\frac{{s}_{BA}^{t}}{{{\rm{\Sigma }}}_{gh,g\ne h}{s}_{gh}^{t}}=\frac{80}{420}=0.190476$ and therefore the *flow* from B to A is $M\frac{{s}_{BA}^{t}}{{{\rm{\Sigma }}}_{gh,g\ne h}{s}_{gh}^{t}}=70\times 0.190476=13.3333$, shown in Table 3 as 13.3.

**Table 3:** Estimated bilateral migration flows from the approach of Dennett (2016) based on rates and migrant stock data from Table 1 and an assumption of 70 total migration flows during the period.

Origin (*t*)	Destination (*t* + 1)				
	A	B	C	D	Sum
A		1.7	1.7	0	3.3
B	13.3		1.7	15	30
C	5	1.7		6.7	13.3
D	10	11.7	1.7		23.3
Sum	28.3	15	5	21.7	70

### Demographic accounting

Demographic accounting methods to estimate bilateral migration flows have two distinguishing characteristics. First, they use the approach of Abel (2013) to rearrange the tables of bilateral migrant stocks, such as in Table 1, into an array of birthplace-specific migration flow data with missing cell values but known marginal values^7^. Second, they control for changes from births and deaths in migrant stocks over the unit interval. We discuss each of these in turn.

#### Demographic accounting, minimisation

Migration flows can be detected from the bilateral stocks in Table 1 from differences in the number of people moving to different places of residence, i.e. moving across columns. Moves across rows are not possible as people’s birthplace (row) remains fixed. Thus, when bilateral migrant stock data from two successive points in time have matching row totals they can be presented as birthplace-specific origin–destination migration flow tables with known margins as shown in the bold typeface in Table 4. In each birthplace-specific flow table, the marginal sums correspond to stock data; the row (origin) sums are from the distribution of migrant stocks at the beginning of the period, and the column (destination) sums are from the distribution of migrant stocks at the end of the period. The remaining cells of each table are unknown, but are imputed using a variety of methods discussed later. For example, in Table 4, in the second sub-table for place of birth B, the last column on the right, headed “Sum” is exactly the transpose of row B from Table 1, left side, showing data at time *t*; and, in Table 4, in the second sub-table for place of birth B, the last row at the bottom, labelled “Sum” exactly equals row B from Table 1, right side, showing data at time *t* + 1.

**Table 4:** Estimated birthplace-specific origin-destination flows under a quasi-independence model based on changes in hypothetical migrant stock data (shown in bold font) and a maximising assumption for the number of stayers on the diagonal cells (shown in italic font).

Birthplace	Origin (*t*)	Destination (*t* + 1)				
		A	B	C	D	Sum
A	A	*95*	0	2.5	2.5	**100**
	B	0	*5*	2.5	2.5	**10**
	C	0	0	*10*	0	**10**
	D	0	0	0	*0*	**0**
	Sum	**95**	**5**	**15**	**5**	**120**
B	A	*75*	5	0	0	**80**
	B	0	*180*	0	0	**180**
	C	0	5	*5*	0	**10**
	D	0	35	0	*55*	**90**
	Sum	**75**	**225**	**5**	**55**	**360**
C	A	*30*	0	0	0	** 30**
	B	7.1	*0*	0	2.9	** 10**
	C	17.9	0	*115*	7.1	** 140**
	D	0	0	0	*40*	** 40**
	Sum	** 55**	**0**	**115**	**50**	** 220**
D	A	*35*	0	5.4	19.6	** 60**
	B	0	*25*	9.6	35.4	** 70**
	C	0	0	*10*	0	** 10**
	D	0	0	0	*160*	**160**
	Sum	** 35**	**25**	**25**	**215**	**300**

Unlike our dummy data in Table 1, in which the column headed “Sum” in the left table is identical to the column headed “Sum” on the right, reported bilateral migrant stock data do not have matching row totals at different times. Apart from measurement errors, the differences are predominantly due to demographic changes from births and deaths over the period. Commonly the numbers of births and deaths of the total population (natives and migrants) are known for each place of residence. These counts can be used to modify the migrant stock tables to reduce differences in row sums when estimating bilateral migration flows. Typically, each migrant stock at the beginning of the period is adjusted by proportionally reducing each stock by the total number of deaths in each place of residence. Death rates are assumed to be the same for foreign-born and native populations in the absence of global measures of mortality rates by nativity that could enable estimation methods to adjust for known heterogeneity. The migrant stocks at the end of the period are adjusted by subtracting the number of births to the native-born populations^7^ in each place of residence from the diagonal elements.

In greater detail, native born populations – the diagonal cells in migrant stock tables - may increase over the period from return migration and new babies. New babies can come from native or foreign-born mothers (i.e. from a migrant stock that is off-diagonal in the stock table). Regardless of their mother’s nativity, new babies increase the native population and hence are counted in the diagonal cell of the stock table. If we do not control for the number of births over the period (by subtracting the total number of births from the diagonal elements), then when we estimate flows from the stock differences (differences in both native and foreign-born) we obtain much higher levels of return migration than expected.

While these controls for demographic changes over the period ensure modified bilateral stock tables with smaller differences in row totals, they do not ensure a perfect match. A match is required to allow successive bilateral migrant stocks to have the same row totals, which permits the migration flows to be estimated. To close the small difference, two approaches have been proposed to further adjust the bilateral migrant stocks.

Abel (2013) proposed estimating the number of migrants from each place of birth, in each place of residence, that leave to places of residence outside the migration system^7^. For example in the hypothetical data of Table 1, this would involve estimated counts of migrants to places of residence outside of the four (A to D) where data are available. The values of these estimated flows to places of residence outside of the migration system are derived so that they sum to the residuals of the difference in row sums between the migrant stock tables at times *t* and *t* + 1. This approach creates an *open* demographic accounting system where persons can move to or from countries beyond the set of those in the input bilateral migrant stock tables.

By contrast, Abel and Sander (2014) proposed scaling each migrant stock table so that the row sums matched the mid-point of their difference, whilst the column totals remained unchanged^8^. This approach creates a *closed* demographic accounting system where all persons either move, do not move, are born or die in the same set of countries.

Our estimates apply both the open and closed demographic accounting methods to produce two different sets of estimates. Both of these estimates use the following minimisation approach to estimate the missing bilateral migration flows, given the marginal totals from the different demographic accounting systems.

Given matching row sums in two successive bilateral migrant stock tables, the cell value *m*_*ijk*_ represents the migration flows from origin *i* to destination *j* for birthplace *k*, where *i*, *j*, *k* = 1, 2, …, *R*, with known margins ${m}_{i+k}={{\rm{\Sigma }}}_{j}{m}_{ijk}$ for row sums and ${m}_{+jk}={{\rm{\Sigma }}}_{i}{m}_{+jk}$ for column sums. The cell values *m*_*ijk*_ can be estimated as:

$${\rm{l}}{\rm{o}}{\rm{g}}\,{m}_{ijk}=\{\begin{array}{cc}{\rm{l}}{\rm{o}}{\rm{g}}\,{\alpha }_{ik}+{\rm{l}}{\rm{o}}{\rm{g}}\,{\beta }_{jk}+{\rm{l}}{\rm{o}}{\rm{g}}\,{\gamma }_{ijk} & {\rm{i}}{\rm{f}}\,i\ne j,\\ \mathop{min}\limits_{ijk}\{log\,{m}_{i+k},\,{\rm{l}}{\rm{o}}{\rm{g}}\,{m}_{+jk}\} & {\rm{i}}{\rm{f}}\,i=j,\end{array}$$

The diagonal cells’ values are set to their highest possible value, which is the minimum of the corresponding row and column marginal sums. These imputed diagonal values are shown in italic font in Table 4. The off-diagonal cells are estimated based on a quasi-independent log-linear model, where *α*_*ik*_, *β*_*jk*_ and *γ*_*ijk*_ are estimated using an iterative proportional fitting procedure^15^ given the row and column sums and diagonal elements. The final estimates from the algorithm are shown in off-diagonal cells of Table 4. Given the full set of *m*_*ijk*_ estimates, origin-destination migration flows can be obtained by summing over birthplaces:

$${y}_{ij}=\left\{\begin{array}{ll}{m}_{ij} & {\rm{if}}\,i\ne j\\ 0 & {\rm{if}}\,i=j\end{array}\right.$$

where ${m}_{ij+}=\sum _{k}{m}_{ijk}$. The estimates from the dummy data are shown on the left side of Table 5.

**Table 5:** Estimated bilateral migration flows from demographic accounting approaches. On the left side, the estimated migration flows are based on the stayer maximisation assumption used in Abel (2013) and Abel (2018). On the right side, the estimated migration flows are based on the Pseudo-Bayesian estimation method of Azose and Raftery (2018).

Minimisation						Pseudo-Bayes					
Origin (*t*)	Destination (*t* + 1)					Origin (*t*)	Destination (*t* + 1)				
	A	B	C	D	Sum		A	B	C	D	Sum
A		5	7.9	22.1	35	A		12	11.3	27.9	51.2
B	7.1		12.1	40.7	60	B	13.5		12.5	45.9	71.9
C	17.9	5		7.1	30	C	21.5	5.3		11.5	38.4
D	0	35	0		35	D	6.2	39.5	4.6		50.3
Sum	25	45	20	70	160	Sum	41.2	56.9	28.4	85.3	211.7

#### Demographic accounting, pseudo Bayesian

As the diagonal elements of *m*_*ijk*_ represent the maximum number of stayers in each country (people with the same origin and destination), the estimated off-diagonal elements are the minimum migration flows required to match the changes in migrant stocks. Azose and Raftery (2018)^11^ addressed this possibly undesirable feature by averaging the minimum flow estimate and an alternative array of migration flows, *z*_*ijk*_, based on an independent log-linear model, without terms for diagonal cells of the array:

$${\rm{log}}\,{z}_{ijk}={\rm{log}}\,{\alpha }_{ik}+{\rm{log}}\,{\beta }_{jk}$$

The *α*_*ik*_ and *β*_*jk*_ terms are estimated using an iterative proportional algorithm given the known marginal totals for each birthplace-specific flow table. Estimates from this model are shown in Table 6, where the known marginal constraints are shown in bold.

**Table 6:** Estimated birthplace-specific origin-destination flows under the independence model based on changes in migrant stock data from Table 1 (shown in bold font).

Birthplace	Origin (*t*)	Destination (*t* + 1)				
		A	B	C	D	Sum
A	A	79.2	4.2	12.5	4.2	**100**
	B	7.9	0.4	1.3	0.4	** 10**
	C	7.9	0.4	1.3	0.4	** 10**
	D	0	0	0	0	** 0**
	Sum	** 95**	** 5**	** 15**	** 5**	**120**
B	A	16.7	50	1.1	12.2	** 80**
	B	37.5	112.5	2.5	27.5	**180**
	C	2.1	6.3	0.1	1.5	** 10**
	D	18.8	56.3	1.3	13.8	** 90**
	Sum	** 75**	** 225**	** 5**	** 55**	**360**
C	A	7.5	0	15.7	6.8	** 30**
	B	2.5	0	5.2	2.3	** 10**
	C	35	0	73.2	31.8	**140**
	D	10	0	20.9	9.1	** 40**
	Sum	** 55**	** 0**	** 115**	** 50**	**220**
D	A	7	5	5	43	** 60**
	B	8.2	5.8	5.8	50.2	** 70**
	C	1.2	0.8	0.8	7.2	** 10**
	D	18.7	13.3	13.3	114.7	**160**
	Sum	** 35**	** 25**	** 25**	** 215**	**300**

The estimated origin-destination migration flows are a weighted average of the estimates of minimum migration flows and those under the independence model:

$${y}_{ij}=\{\begin{array}{cc}{m}_{ij+}w+{z}_{ij+}(1\,-\,w) & {\rm{i}}{\rm{f}}\,i\ne j\\ 0 & {\rm{i}}{\rm{f}}\,i=j\end{array}$$

Azose and Raftery (2018) estimated *w* = 0.87 using a pseudo-Bayesian approach to minimise a cost function of the logged differences between estimated outward migration flow rates with equivalent estimates of European migration flows between 2002 and 2008 from the IMEM project^16^. Their application of the method used the closed demographic accounting system to obtain marginal totals for the birthplace-specific flow table of *m*_*ijk*_ and *z*_*ijk*_. Estimates using the same value of *w* for the dummy data are shown on the right side of Table 5.

## Estimates of Bilateral International Migration Between all Pairs of 200 Countries

To estimate bilateral migration flows from changes in bilateral migrant stocks for all countries between 1990 and 2015, we obtained and cleaned input data (Fig. 1). Demographic and bilateral migrant stock measures came from the United Nations Population Division (UNPD) as a sequence of bilateral migrant stock data covering all pairs of 232 countries for the mid-years of 1990, 1995, 2000, 2005 and 2015^17^. At the time of writing (February 2019), this was most up-to-date set of bilateral migrant stocks available for all countries. Data were primarily based on birthplaces reported in censuses and population registers provided by national statistical institutes. The UNPD adjusted data to include available refugee statistics. When recorded data did not align to the mid-years at five-year intervals, the UNPD extrapolated values based on the change in the overall population size. For countries or areas without data, the UNPD used a similar country or group of countries to estimate missing bilateral stocks.

The demographic accounting flow estimation methods require population totals at the beginning and end of each time period, and births and deaths over each interval. Further, the migration rates method requires net migration estimates during each period. Estimates of each of the measures were obtained from the 2017 version of the UNPD *World Population Prospects* (WPP2017)^18^.

Of the 232 countries in the migrant stock data, only 200 had complete estimates of other demographic measures such as births and deaths. A full list of the countries used is given in Supplementary File 1. We excluded all countries that lacked complete information. All of the 32 excluded countries had populations below 100,000.

G.J.A. developed the migest R package^19^ to calculate each of the estimation methods outlined in the previous section (namely, a. stock differencing approach based on dropping zeros, b. stock differencing approach reversing negative estimate flows, c. migration rates approach, d. demographic accounting using an open system and minimisation approach to estimate missing flows, e. demographic accounting using a closed system and minimisation approach to estimate missing flows and f. demographic accounting using a closed system and pseudo-Bayesian approach to estimate missing flows). These routines were applied to clean sets of complete data for 200 countries five times to estimate bilateral migration flows for each five-year period between 1990 and 2015. The R code for cleaning the data and estimation is briefly described in the code availability section.

**Fig. 2:**
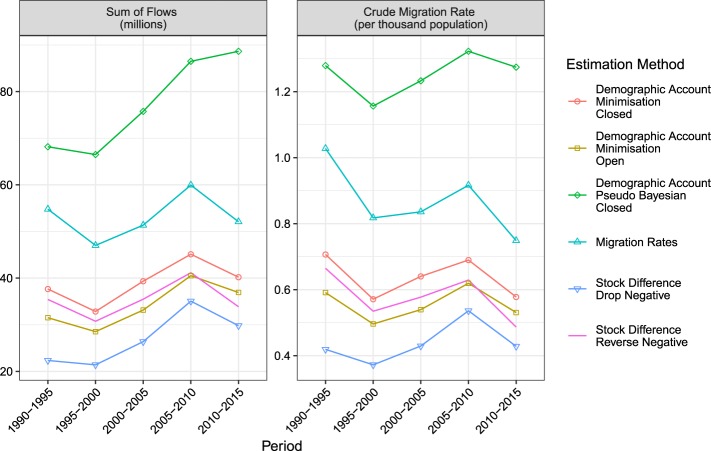
Total migration flows (in millions) and their corresponding crude migration rates (migrants per thousand people in the population) for five-year periods between 1990 and 2015 based on six flow estimation methods.

**Fig. 3:**
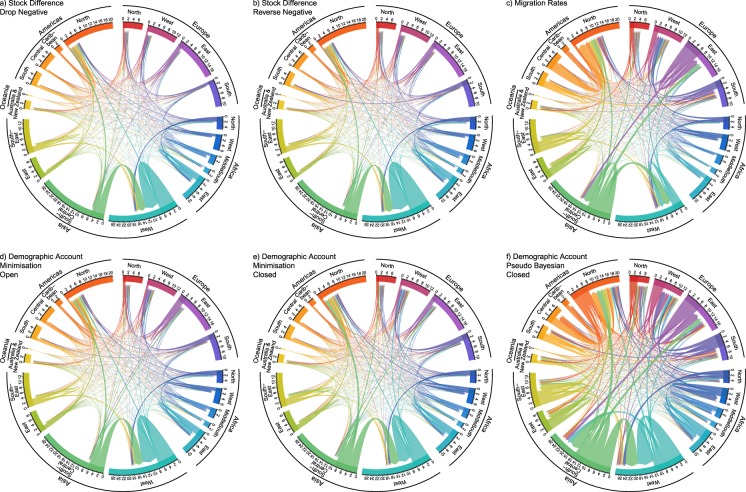
Chord diagrams of estimated migration flows during 2010–2015 based on six methods. Direction of the flow is indicated by the arrowhead. The size of the flow is indicated by the width of the arrow at its base. Numbers on the outer section axis, which give the size of migration flows, are in millions of individuals per five-year period. Sector axis limits are based on maximums over all estimation methods and all five-year periods.

The total migration flows and their corresponding crude migration rates, obtained by dividing the total migration flows (summed over all pairs of countries in both directions) by the total population of all countries at the beginning of the period, are shown in Fig. 2. Summary statistics by estimation method are shown in Table 7. The bilateral migration patterns during the most recent period (2010–2015), aggregated to the United Nations regions, are shown in Fig. 3. To facilitate comparisons, the margins of each regional sector of the chord diagram plot are set to their maximum values over all time periods and estimation methods. Plots were produced using the circlize R package^20^. Animations of the changes in regional bilateral patterns for each estimation method, plotted with both fixed and non-fixed margins, can be found on Figshare^21^.

**Table 7:** Summary statistics for bilateral migration flows over five-year periods between 1990 and 2015 based on six flow estimation methods. All numbers except the Proportion Zero are in units of individuals per five years. N is the number of migration corridors modelled: 200 countries of origin × 200 countries of destination × 5 quinquennial intervals.

	Stock Difference Drop Negative	Stock Difference Reverse Negative	Migration Rates	Demographic Accounting Minimisation Open	Demographic Accounting Minimisation Closed	Demographic Accounting Pseudo Bayesian Closed
N	200,000	200,000	200,000	200,000	200,000	200,000
Minimum	0	0	0	0	0	0
Median	0	0	0	0	0	0
Mean	675	883	1,326	853	976	1,928
Maximum	2,763,183	2,763,183	3,492,367	2,865,526	2,899,536	3,918,816
Std. Dev.	15,322	17,770	22,710	17,470	16,926	25,492
Proportion Zero	0.82	0.79	0.76	0.72	0.7	0.53
Mean (non-zero)	3,788	4,112	5,513	3,025	3,206	4,096
Std. Dev. (non-zero)	36140	38167	46061	32801	30564	37039

## Data Records

The international migration flow estimates described in this article, between all pairs of 200 countries listed in Supplementary File 1, are publicly and freely available through Figshare^21^. The estimates are stored in a single file with separate rows for each migration corridor - period combination (200 origins × 200 destinations × 5 periods = 200,000) and separate columns for estimates based on each method described above: sd_drop_neg, sd_rev_neg for the estimates using the stock differencing approaches; mig_rate for estimates using the migration rates approach; and da_min_open, da_min_closed and da_pb_closed for estimates using the demographic accounting approaches.

## Technical Validation

We compared the estimated bilateral migration flows with two data sets: bilateral and total migration flows from selected countries reported by the United Nations^22^; and net migration flows for all countries from WPP2017. The first data set is based on numbers of migrants sent and received by 45 countries using a variety of definitions of migration events and different concepts to determine their origin and destination. We used the reported migration flows between 1990 and 2015 based on previous or next place of residence for the total population, for which there are 32,698 one-directional migration corridors-period combinations that correspond with our estimated bilateral migration flows.

For each of our 200 countries, for each five-year period, for each of the six methods of estimation, we computed an estimated net migration as the difference between the sum of immigrants from all other countries, minus the sum of all emigrants to all other countries (among the 200 countries in our study). A positive net migration therefore meant more immigration than emigration. We compared each estimated net migration with the corresponding net migration flows from the most recent WPP. The WPP2017 net migration data are based on a combination of net residuals from population accounting and official migration statistics, where they exist. WPP2017 estimates reflect demographic accounting residuals, net migration counts (where available), accumulated statistical errors from population, fertility and mortality estimates, and adjustments the UNPD carried out to ensure that global net migration is zero in each five-year period. The WPP2017 net migration estimates are available for all pairs of 200 countries and periods where we have migration flow estimates.

### Calculation of equivalent reported flow

Reported migration flow data collected by the UN are for single years, whereas the estimated flows are based on the migration flows over a five-year period. Thus, for a given five-year period in a migration corridor there are potentially five reported migration flows to compare with a single estimated value. To provide an approximate guide to the level of reported migration flows in each five-year period we multiplied the average annual migration flow in each corridor and period by five.

We believe this modified reported flow should be highly correlated with a good estimated flow. However, we do not believe that the two numbers will match precisely as there are a range of measurement issues that prohibit an exact match. For example, national statistical institutes use different criteria to define how long a new entrant to the country must stay to qualify as a migrant. In some countries, a new entrant must declare an intention to settle permanently. In others, a stay of one year, six months, three months, or one month qualifies a new entrant as a migrant. These definitions may boost or dampen counts of migration flows along specific corridors.

We do not adjust the UNPD WPP2017 net migration data for the validation, as both the time period and the measurement of migration match those of the estimated flows from all methods. Explicitly, the UN net estimates and the estimates from our application of any of the methods have corresponding time periods (mid-year to mid-year over 5-year intervals) and both count migrant transitions (based on migrants’ location at the beginning and end of the interval) rather than the number of moves during the interval.

### Validation measures

Due to the problems in comparing the reported annual migration flows with estimated five-year flows, we do not measure validity by accuracy compared to a ‘true’ observed value, as would be measured for example by the mean absolute error or mean squared error. Instead, we use Pearson’s correlation between the estimated five-year flows and the modified reported flows to measure the validity of estimates. Hence, we avoid the need for an accurate multiplier for the average of the annual reported flows because the correlation between two positive variables is invariant when either variable is multiplied by a positive constant, or both are.

We assess the correlation between the estimated migration flows and available reported data by using six common measures of migration (Table 8). These six measures reflect possible applications of migration flow estimates to monitor trends (e.g. counts), explore patterns and migration drivers in explanatory models (e.g. logarithm of counts), as weights to detect migrant network structures (proportions), or baseline measures for inputs into population projection models (immigration and emigration rates or net migration counts).

**Table 8:** Migration measures used for validation. In this table, *y*_*ij*_ is a general term for a bilateral migration flow from either an estimation method or the equivalent reported data. All numbers except the Proportion are in units of individuals per five years.

Measure	Measure Calculation (Single Period)	Reported Flow Observations (All Periods)	Reported Flow Data Source
Count	*y*_*ij*_	32,698	International migration flows to and from selected countries: The 2015 revision. United Nations Population Division
Natural Logarithm of Count	log*y*_*ij*_	32,698	International migration flows to and from selected countries: The 2015 revision. United Nations Population Division
Proportion	$\frac{{y}_{ij}}{{\sum }_{j}{y}_{ij}}\,{\rm{o}}{\rm{r}}\,\frac{{y}_{ij}}{{\sum }_{i}{y}_{ij}}$	32,698	International migration flows to and from selected countries: The 2015 revision. United Nations Population Division
Emigration Rate	$\frac{{{\rm{\Sigma }}}_{i}{y}_{ij}}{{p}_{i}}$	155	International migration flows to and from selected countries: The 2015 revision. United Nations Population Division
Immigration Rate	$\frac{{{\rm{\Sigma }}}_{i}{y}_{ij}}{{p}_{j}}$	172	International migration flows to and from selected countries: The 2015 revision. United Nations Population Division
Net Migration Count	$\sum _{i}\,{y}_{ij}\,-\,\sum _{j}{y}_{ij}$	1000	World Population Prospects: The 2017 Revision. United Nations Population Division

The first measure is the count (number of individuals per five years) of a migration flow. Estimates that are statistically independent of the reported flows will have a sample correlation not far from zero, and estimates that are only weakly related to reported flows will have a low correlation.

The second measure is the natural logarithm of the count. Estimates that are randomly variable percentages of the reported flows will have low correlation.

The third measure is the proportion of migrants, where the denominator is either the total inflow or outflow over the period depending on which reported data source (receiving or sending data) is used. These three measures can be calculated for 32,698 corresponding equivalent reported bilateral flows. Of these, 5,134 observations are based on 2,567 origin-destination-period combinations where both sending and receiving data are available.

The fourth and fifth measures are the emigration rate from a source country and the immigration rate to a destination country, respectively. They divide the flows out of (emigration) and into (immigration) a country by the population of either the origin or the destination. The population sizes in the denominator are the corresponding population sizes in WPP2017 at the beginning of the five-year period.

The sixth measure is the net migration count. The WPP2017 reported net migration counts for our 200 countries in all five periods, enabling us to compare 1,000 estimates and reports.

### Overall agreement

**Fig. 4:**
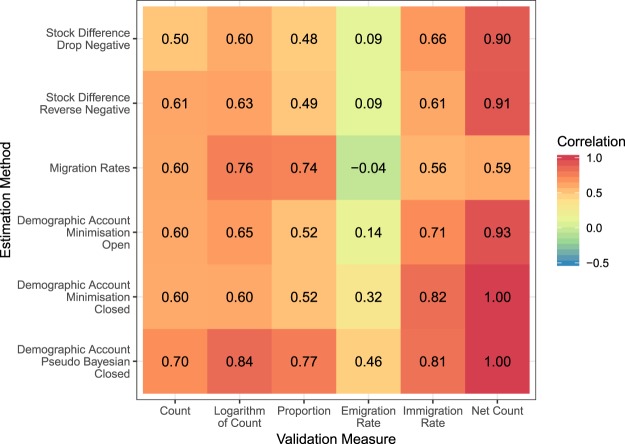
Correlations between estimated migration flows during five-year periods from 1990 to 2015 from six estimation methods with equivalent reported migration flows.

For each of these six measures, we calculated the Pearson correlation between the estimated flows and the equivalent reported flows (Fig. 4). For all bilateral measures (count, logarithm of count, proportions), the estimates based on Azose and Raftery (2018) method have the highest correlations (bottom row of Fig. 4). The correlation for the logarithm of count measure is at least as strong as or stronger than the non-transformed counts over all estimation methods (correlations in column 2 of Fig. 4 are greater than or equal to correlations in column 1).

The correlations of the estimated and reported emigration rates (column 4) are lower for all methods than the correlations of all other migration measures. The two stock differencing and migration rates methods have close to zero correlation between their emigration rate estimates and the reported emigration rates. Correlations of the immigration rates are higher for the closed demographic accounting method of Abel (2018) and Azose and Raftery (2018). These two methods produce estimates that are perfectly correlated with reported net migration counts in all countries because both methods use the scaling technique of Abel and Sander (2014). This technique implicitly constrains the estimated bilateral migration flows sum to net migration by adjusting migrant stock totals so that their differences over the total population follow the demographic accounting equation. Other estimation methods that do not use this technique, except for migration rates method (row 3), have higher correlations (column 6) between their net migration counts and the reported data than correlations using other migration measures.

**Fig. 5:**
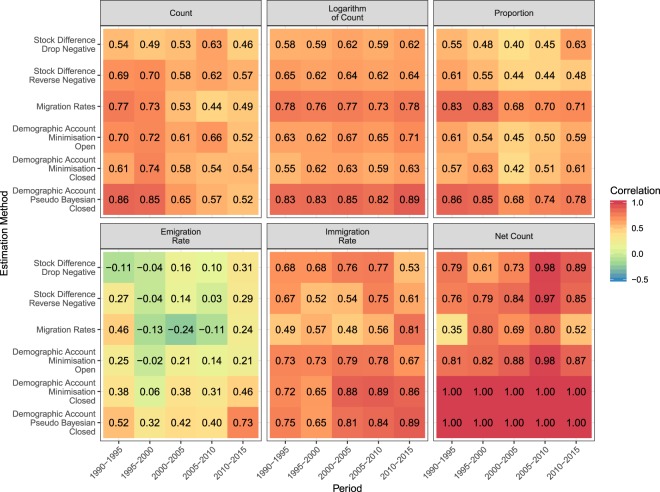
Correlations in five-year periods between estimated migration flows from six alternative estimation methods with equivalent reported migration flows.

For greater temporal resolution, we calculated the correlation for each estimation method and each validation measure in each time period (Fig. 5). For many of the methods, the migration count correlations appear to decline in more recent five-year periods, while the correlations in the logarithm of counts are relatively stable or slightly increasing. Proportion correlations for most methods fall to lows during 2000–2005 and then increase. Correlations in the emigration rates are generally lower and vary more over time than the correlations for the immigration rates. The highest correlations between the reported and estimated immigration rates occur in later periods based on closed demographic accounting methods (bottom right corner of the panel for Immigration Rate in Fig. 5). The correlations between the reported net migration counts and the estimates peak during 2005–2010, with the exception of the estimates from the closed demographic accounting methods that perfectly correlated in every period (next-to-last column of the panel for Net Count in Fig. 5).

**Fig. 6:**
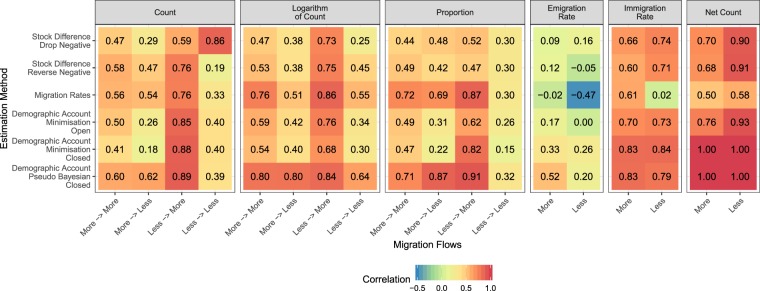
Correlations between estimated migration flows from six estimation methods with equivalent reported migration flows by development level of country.

We also analysed the effects of the level of development (more or less developed) on the correlations of estimated flows with the reported data for each validation measure using the development level of the origin and destination in the United Nations World Population Policy Data^23^ (Fig. 6). The development category for each country is listed in the un_group column of the country list in Supplementary File 1. Over all periods, there are 471 equivalent reported migration flows between less developed countries, 6,667 between more developed countries, 10,580 from more to less developed countries and 12,413 from less to more developed countries. Only five countries (Armenia, Azerbaijan, Cyprus, Kazakhstan and Kyrgyzstan) reported flows between less developed countries. The correlations for net migration counts were based on all countries (155 less developed, 45 more developed) because the UNPD generated net migration figures regardless of the availability of supporting data.

The correlations of bilateral migration measures with equivalent reported data are highest for migration flows from less to more developed countries for all estimation methods, with the exception of the counts based on the drop negative stock differencing approach (where the correlations between reported and estimated flows to and from less developed countries are highest). The correlations of estimated emigration rates with equivalent reported flows are higher for migration flows from more developed countries for all estimation methods except Stock Difference Drop Negative. There was less uniformity for the immigration rates, where higher correlations were found for flows into more developed countries for only the estimates from the migration rates and pseudo-Bayesian approach. For the four estimation methods without the implicit net migration constraint, all had higher correlation of net migration counts with reported data for less developed countries.

These validation exercises provide new insights into global migration flow estimates. First, the correlation between the reported data and the estimates varies widely among different migration measures. For example, correlations of emigration rate estimates with reported data are lower than correlations of immigration rate estimates with reported data. Second, the correlations between the estimates and reported data vary widely in both time and space. Third, over most migration measures, estimates based on closed demographic accounting methods, especially those using the pseudo-Bayesian method, have the highest correlations.

## Usage Notes

The estimates in this paper apply six estimation methods to the most recently available bilateral migrant stock data. The estimation methods based on migration rates and demographic accounting require additional information on demographic measures. Consequently, estimates that require these additional data are susceptible to errors propagated from inaccuracies in the stock and demographic data. As Rees (1980) suggested, demographic accounting methods such as those used here help users to identify consistencies, check for inadequacies, and match data with a conceptual model^24^.

The six methods of estimation used here share some limitations. All methods estimate that at least half of the bilateral migration flows are zero (Table 7). In addition, all estimates generate the number of migration transitions (i.e. the number of people in country *i* at the beginning of the period and country *j* at the end of the period); they do not take account of additional migration events to third countries or return moves during the period. Estimates based on stock differences and the demographic accounting methods by Abel (2013) and Abel (2018) produce minimum estimates of migration flows. Estimates based on stock differencing or migration rates ignore changes in stocks that might have occurred from deaths of migrants outside their country of birth. In cases with large elderly migrant stocks, deaths of migrants outside their country of birth could lead to overestimating migration flows.

Our validations of the bilateral migration and rate measures were carried out only where migrant flow data were reported, mainly from rich western countries; if and when migrant flow data become available from less developed countries, the validity of these measures may be different.

## Supplementary Information

### ISA-Tab metadata file

Download metadata file

### Supplementary information

Supplementary File 1

## Data Availability

All estimation routines to produce the open-access estimated five-year migration flows for all pairs of 200 countries between 1990 and 2015 are in the migest R package available on CRAN^19^. The formatted data and R code for the application of the functions are publicly available on Figshare^21^. They are 1) a tidy version of the United Nations bilateral migrant stock data, demographic period measures for net migration, births and deaths and demographic population totals; 2) an R script that given the three imported data sets a) cleans each to find the countries with complete input data; b) calculates the native population for the diagonal elements of the migrant stock data required for some estimation methods; and c) estimates bilateral migration flows for each method and period combination.
